# Ovarian Cancer Cell Adhesion/Migration Dynamics on Micro-Structured Laminin Gradients Fabricated by Multiphoton Excited Photochemistry

**DOI:** 10.3390/bioengineering2030139

**Published:** 2015-07-16

**Authors:** Ruei-Yu He, Visar Ajeti, Shean-Jen Chen, Molly A. Brewer, Paul J. Campagnola

**Affiliations:** 1 Department of Biomedical Engineering, University of Wisconsin-Madison, Madison, WI 53717, USA; E-Mails: imreyu@gmail.com (R.-Y.H.); ajeti@wisc.edu (V.A.); 2 Department of Engineering Science, National Cheng Kung University, Tainan 701, Taiwan; E-Mail: sheanjen@mail.ncku.edu.tw; 3 Department of Obstetrics and Gynecology, University of Connecticut Health Center, Farmington, CT 06030, USA; E-Mail: mbrewer@uchc.edu

**Keywords:** ovarian cancer, ECM, haptotaxis, contact guidance, morphology, cytoskeleton

## Abstract

Haptotaxis, *i.e.*, cell migration in response to adhesive gradients, has been previously implicated in cancer metastasis. A better understanding of cell migration dynamics and their regulation could ultimately lead to new drug targets, especially for cancers with poor prognoses, such as ovarian cancer. Haptotaxis has not been well-studied due to the lack of biomimetic, biocompatible models, where, for example, microcontact printing and microfluidics approaches are primarily limited to 2D surfaces and cannot produce the 3D submicron features to which cells respond. Here we used multiphoton excited (MPE) phototochemistry to fabricate nano/microstructured gradients of laminin (LN) as 2.5D models of the ovarian basal lamina to study the haptotaxis dynamics of a series of ovarian cancer cells. Using these models, we found that increased LN concentration increased migration speed and also alignment of the overall cell morphology and their cytoskeleton along the linear axis of the gradients. Both these metrics were enhanced on LN compared to BSA gradients of the same design, demonstrating the importance of both topographic and ECM cues on the adhesion/migration dynamics. Using two different gradient designs, we addressed the question of the roles of local concentration and slope and found that the specific haptotactic response depends on the cell phenotype and not simply the gradient design. Moreover, small changes in concentration strongly affected the migration properties. This work is a necessary step in studying haptotaxis in more complete 3D models of the tumor microenvironment for ovarian and other cancers.

## 1. Introduction

In 2013 there were an estimated 22,000 new cases of ovarian cancer in the United States and 15,000 deaths (Cancer Facts and Figures 2013, American Cancer Society, Database), where the five-year mortality rates for stage 3 and 4 cancers were ~80%–90%. There remains a need for the development of more efficacious treatments to improve these outcomes. Achieving all these goals require a better understanding of the underlying basic science of the tumor microenvironment (TME), for example, in terms of alterations of cell migration dynamics [1,2,3,4,5,6]. This is especially important for ovarian cancer as migration is highly regulated in the normal ovary.

It is generally accepted that metastasis often occurs from exfoliation into ascites, and then by reattachment in the intraperitoneal cavity (IP), interacting with mesothelial cells, and then by invasion into the local stroma [7]. These dynamics are generally regulated by matrix binding integrins (e.g., β_1_), matrix degrading proteinases (MMPs), and cell-cell adhesion molecules (cadherins). Treatments targeted at such species that govern migration have been investigated but have not yet led to positive clinical outcomes [1,2,3,4,5,6]. This may have arisen due to incomplete mechanistic knowledge of the cell dynamics and their interaction with the extracellular matrix (ECM). While mis-regulation of migration is generally associated with cancer invasion and metastasis, single genes have not typically been associated with enhanced migration and it has been speculated that different species act in concert [3]. Moreover, specific phenotypes within a heterogeneous population of the cell behavior (even with the same cell type) may contribute differentially to bulk measurements. In addition to gene expression, the resulting behavior will also likely depend on the local 3D composition and architecture [3]. Focusing on cell–matrix interactions under well-controlled *in vitro* conditions, *i.e.*, investigating the cell response, to ECM protein composition and concentration, and also morphology may therefore yield new insight into the relationship between migration and cancer.

A variety of 2D and 3D *in vitro* techniques have been employed to study cell migration, each with its own strengths and weaknesses. For example, transwell or Boyden chambers have been used to study migration/invasion [8,9,10,11,12], and while the coated membranes present some of the relevant biochemical cues, these 2D substrates are not faithful models of the native ECM as the nano/microtopography and biochemistry are important for cell adhesion, phenotype, and migration [13]. Matrigel is often used as a biomimetic ECM substrate but the actual composition is not well known and not reproducible. There remains a need for better models of the basement membrane to study cancer cell migration dynamics.

In addition to protein composition and morphology, cancer cell migration in response to insoluble gradients, *i.e.*, haptotaxis, may also be important [14,15,16]. While not as well-studied as chemotaxis (response to soluble stimuli), several techniques have also been employed to create physical, insoluble (stiffness and concentration) gradients for studying cell motility. Approaches such as microfluidics [17,18,19,20,21,22] and microcontact printing have been developed for creating 2D gradients in a variety of geometries [23,24]. It would be further advantageous to create concentration gradients with 3D features directly from native full-length ECM proteins assembled with nano/microscale topographic features as binding domains on this sizescale are recognized by cell integrins. This approach would afford decoupling the roles of pure contact guidance from the combination of ECM cues with contact guidance through control of both composition and topography.

To achieve this goal, we created covalently linked micro-structured ECM protein gradients through the use of multiphoton excited (MPE) photochemistry. This photochemical process is analogous to two-photon excited fluorescence microscopy (TPEF), where the excitation, and here, the fabrication, is confined to the focal volume, resulting in intrinsic 3D capabilities and concurrently affording sub-micron feature sizes [25,26,27,28,29,30,31,32]. The crosslinked protein concentration achieved by MPE can be well-controlled by tuning the laser exposure and thus can be varied independently from the topography [28,33,34]. For example, we previously used MPE to fabricate fibronectin (FN) and BSA concentration gradients to investigate the adhesion and spreading dynamics of 3T3 fibroblasts both in terms of the resulting morphology and the cytoskeletal response [35]. We considered these gradients to be of dimension 2.5D, *i.e.*, having limited but finite height.

In this paper, we use analogous micro-structured laminin (LN) gradients of differing slope and concentration to study the migration of ovarian cancer cell lines of varying metastatic potential. LN was chosen, as it is one of the two primary components of the ovarian basal lamina. Previously we used “fibers” of constant LN concentration to compare migration speed, where the migration was the result of a combination of contact guidance and ECM cues [36]. Due to the suggested relevance of gradients in metastasis, we extended this work to examine the relationships between speed, cytoskeletal dynamics (stress fiber orientation and focal adhesion distribution), gradient slope and metastatic potential. Here we compared “normal” immortalized epithelial cells (IOSE) as well as three ovarian cancer lines of differing metastatic potential (OVCA433, SKOV-3.ip1, HEY-1 in increasing order). We find that the behavior arises from combined effects of ECM cues and contact guidance, which are separable using these fabricated constructs. We also find that small changes of gradient slope and local concentration can have significant effect on the observed migration. We will show that the shape of the cells also matters in the response to the gradient in terms of speed, directed migration, and cell alignment with respect to the gradient.

## 2. Methods

### 2.1. Fabrication Instrument and Photochemistry

The multiphoton fabrication instrument/microscope has been described in detail previously [26]. Briefly, the multiphoton photochemistry is induced by a femtosecond titanium sapphire laser (Coherent MIRA) that is coupled to a purpose-built laser scanning microscope system mounted on an upright stand (Zeiss Axioskop). The scanning/acquisition software is written in LabVIEW and is freely available upon request. Here two-photon excitation of the water-soluble porphyrin TMPyP was employed at 780 nm with an average power at the focus of 100 mW, using a 0.5 numerical aperture (NA) lens. This activator has a high triplet state quantum yield, and the reactive triplet state creates singlet oxygen, which reacts with proteins creating a radical and ultimately a protein–protein crosslink.

### 2.2. Sample Preparation

The laminin gradients were fabricated on a microscope slide, where a self-assembled organosilane monolayer (octadecyltrichlorosilane; ODTS) is coated with a monolayer of BSA (10 mg/mL) to form the base for the crosslinked LN [28]. As reported previously [34], the BSA is used as a non-specific surface to compare the adhesion dynamics of cells adhered to and away the crosslinked LN. By fluorescence microscopy imaging of the BSA (~1% concentration labeled with Texas Red), we found this background was stable over the 72 h period in which cell adhesion measurements were performed. The fabrication solution consisted of 0.5 mg/mL LN (mouse) and 2.5 mM porphyrin in water and was confined in a small circular rubber chamber seated on top of the self-assembled BSA monolayer [28]. After fabrication, the structures were washed with deionized water, rinsed with PBS pH 7.4 which contained 400 μg/mL penicillin and 400 μg/mL streptomycin under sterile conditions, and kept hydrated for cell plating.

### 2.3. Gradient Design

The overall gradient structures consisted of a series of individual linear gradients where each was 800 μm in length and spaced by 10 microns. This separation is smaller than individual cells and allows interactions with several features simultaneously and has been used previously [34,36]. As determined by higher resolution optical microscopy, this optical setup using 0.5 NA yielded minimum feature sizes of approximately 700 nm in diameter and two microns in height [26]. The gradients were designed such that the point density of the protein fabrication increased linearly from 0.1 point/μm to 1.0 point/μm (higher slope and higher final concentration; referred to hereafter as “high slope gradient”) or 0.01 point/μm to 0.4 point/μm (lower slope and lower concentration; referred to hereafter as “low slope gradient”) while the laser power was maintained at a constant average power of 100 mW at the focus. At the low concentration regions, the crosslinked LN is localized into discrete points, which become continuous at the higher point densities, which occurs at approximately 0.2 point/μm. Higher point densities then result in increased local concentrations with the same topography. The average concentration across the gradients remained linear even where the individual points were discrete. As a control to separate the effects of morphology from ECM binding cues, analogous gradients with the same design parameters were made from BSA.

### 2.4. Cell Culture and Time-Lapse Microscopy

IOSE [37], OVCA433 [37], SKOV-3.ip1 and HEY-1 (from Dr. Susan Huang, MD Anderson Cancer Center) [38] cells were cultured on the 24-plate multiwell plate to confluence with 1:1 Dulbecco’s Modified Eagle’s medium and F12 (GIBCO) supplemented with 10% fetal bovine serum (GIBCO) in a humidified incubator at 37 °C in which the CO_2_ level was maintained at 5%. We used 10% FBS in these studies (both culture and migration measurements) as prior work showed that IOSE cells do not grow well in lower serum levels [37]. The cells were trypsinized and fabricated structures were seeded with ~24,000 cells/mL in culture medium (DMEM/F12) and incubated. The cells were allowed to adhere and spread for approximately 5 h and mineral oil was then added on top of the culture medium to prevent evaporation and keep the cells sterile.

Migration rates were measured on an inverted microscope (Nikon ECLIPSE TE300) equipped with a CCD camera (Watec WAT-902B) and an automatic temperature controller (Warner Instruments TC-324B). The samples were maintained at 37 °C and phase contrast images were acquired with a 4× objective (Nikon CFI Plan Fluor DL) at 10 min intervals for a total period of ~48 h. ImageJ software (http://rsb.info.nih.gov/ij) was used to manually trace the trajectory of individual cells.

The instantaneous speeds of individual cells were determined by measuring the position of the middle of each cell from one frame to the next divided by the 10 min long period between these frames. For this analysis, we divided the migration data into three 200 µm long regions that span the entire length of the gradient. If a cell crossed from one region to the next, the cell tracking and calculation of its migration speed ceased and it was treated as a new cell/track entering the second region. Data were acquired from 3–4 independent measurements and analyzed cumulatively based on the total number of cells.

### 2.5. Dual Staining for Focal Adhesions and F-Actin

For focal adhesion and actin staining, the cells were incubated overnight and fixed for 15 min with 4% formaldehyde. The cells were permeablized with 0.2% triton X-100 and treated with 1% BSA in PBS to block non-specific sites. For the focal adhesion staining, the cells were first incubated with anti-vinculin (1:200, Abcam ab1119) for 1 h, followed by a secondary fluorescent antibody (goat-anti-mouse F(ab)′_2_) IgG conjugated with Alexa Fluor (Invitrogen; Carlsbad, CA, USA) for 45 min. To stain for F-actin, the cells were incubated with Texas Red conjugated phalloidin for 45 min (simultaneously with the IgG secondary fluorescent antibody). Fluorescence images of both the AlexaFluor and the Texas Red channels were recorded with a highly sensitive CCD camera (Andor technology DV887ECS-BV) using a 40 × 0.65 NA objective. The corresponding phase contrast images were recorded sequentially.

ImageJ (http://rsb.info.nih.gov/ij) was used to assess the angular distribution of the F-actin distribution in the cells at different LN concentrations. Circular statistical analysis was used to provide a spatial histogram and circular deviation of the stress fiber orientation over the entire cell with respect to the linear LN axis [39]. Here, the mean resultant of the stress fibers, R, is a measure of orientation, given by:

(1) $$R=\frac{1}{N}\sqrt{\sum_{j=1}^{N}\sin2\theta_{j}+\sum_{j=1}^{N}\cos2\theta_{j}}$$

where *N* is the number of stress fibers, and θ is the angle of the individual stress fibers.

ImageJ was also used to determine the cell morphological properties with respect to the gradient direction. Here each individual cell was modeled as an ellipse, and the elongation ratio (*i.e*., ratio of major to minor axes) and the orientation (angle between the long axis of the ellipse and the LN gradient direction) were measured.

### 2.6. Statistic Analysis

Migration speeds, migration direction, and orientation, and stress fiber average orientation are reported as the mean value with the corresponding standard error for the four cell types. For migration speed and migration direction, pairwise *t*-tests (Origin 7.0; OriginLab Corporation, Northampton, MA) were used to compare the three different cancer cell lines to the IOSE cells (the normal control), as well as to each other. A standard significance level of *p* < 0.05 was used for all the analyses. For cell orientation and stress fiber angular distribution, the Pair-wise Watson *U*^2^ test was used to examine statistical differences in the cell orientation, directed migration, and stress fiber orientation. A standard significance level of *p* < 0.05 was also used for these analyses. The Rayleigh test was used to determine if migration and cell alignment had a defined direction.

## 3. Results

### 3.1. Gradient Design

**Figure 1:**
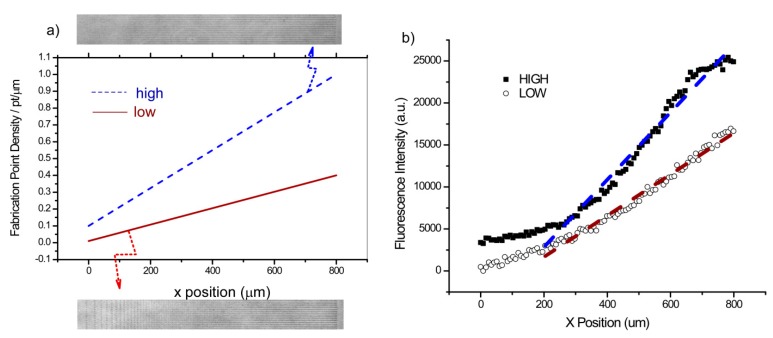
Design of the low and high slope gradients: (**a**) Linear ramp showing the design as a function of point density, along with the resulting phase contrast image of the fabricated gradients. (**b**) The resulting LN immunofluorescence and corresponding linear fit (*R*^2^ > 0.95) representing the relative concentrations.

The resulting morphology and relative concentration range and slopes of the two gradient designs were determined by optical microscopy. Figure 1a shows the design of the two gradients (low slope and high slope; see Methods for details) in terms of linear point density along the long axis. The representative respective phase contrast images of the gradients are show adjacent to the design. The relative concentration of the LN gradients was determined by immunofluorescence, where the structures were incubated with a primary LN antibody followed by a secondary fluorescent antibody. The fluorescence intensity and thus relative concentrations of the two designs are plotted in Figure 1b with the best linear fit.

We ignored the first 200 microns of the gradient where the points are highly discrete and only used the 200–800 µm range. While there is scatter in the fluorescence intensities, the data in each case is well-fit by linear regression (*R*^2^ > 0.95), and the change in slope between the low and high designs was approximately 1.5. The predicted ratio based on the design was 2.3, where the difference may have occurred through a variety of factors. However, it is likely that the upper end of the higher concentration design was in the limit which we have previously defined as the terminal crosslink density [40], where more laser exposure does not result in higher concentration, as all the available reactive sites on the protein have already been utilized. The difference in concentration at the end of each gradient was also approximately a factor of 1.5.

### 3.2. Migration Speed and Directionality

**Figure 2:**
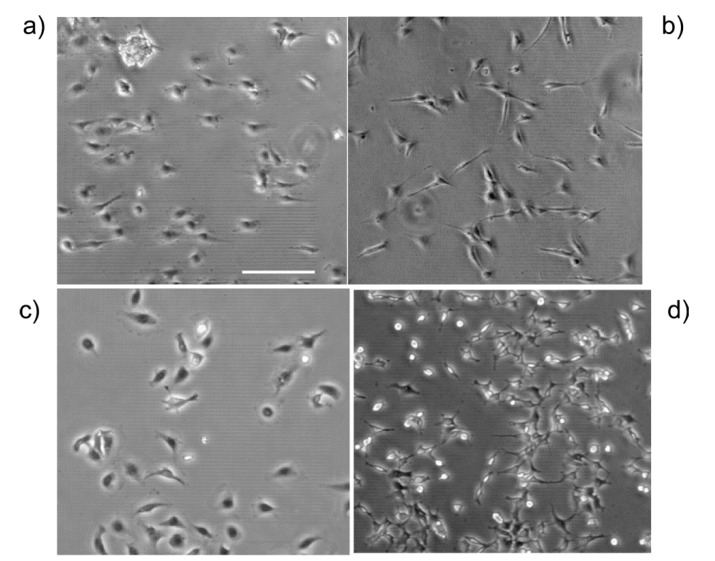
Representative phase contrast images of each cell line on the high slope gradient, where (**a**) IOSE; (**b**) OVCA433; (**c**) SKOV-3.ip1; and (**d**) HEY-1. The gradient increases in concentration from left to right. Scale bar = 200 microns.

A representative phase contrast image of the IOSE, OVCA433, SKOV-3.ip1 and HEY-1 cells on LN gradients is shown in Figure 2.

**Figure 3:**
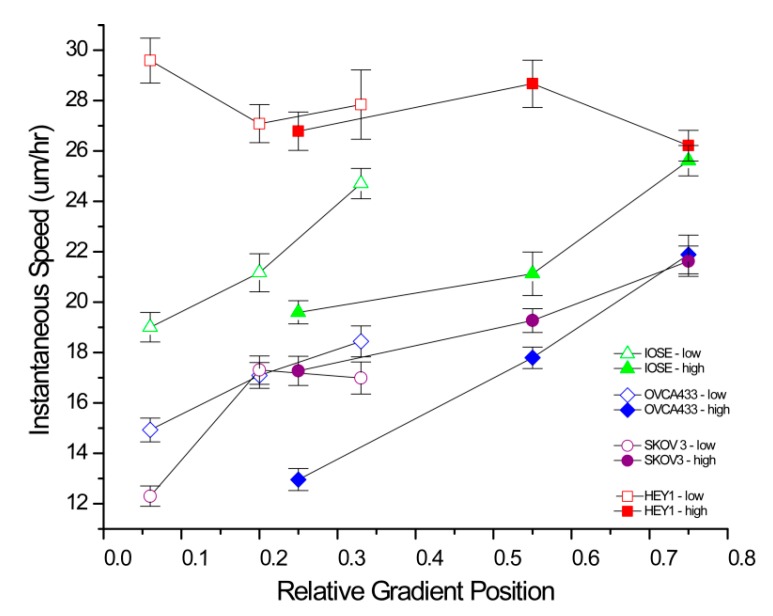
Merged migration speed data for the four cell lines on the low and high slope gradients. The statistical analysis for each cell line is given in Table 1, Table 2, Table 3 and Table 4. The data shows the relative roles of local concentration and slope on the haptotaxis response of the cell lines. Error bars represent standard error.

The averaged instantaneous speeds with the mean error are summarized in Figure 3 for each cell type on the low and high gradient designs, where we break down each into three regions, which we delineate as low, medium and high concentration. We plotted the instantaneous speeds based on the relative position of the gradient where we defined the spread from 0 to 1. Specifically, cell measurements done on “mid” and “high” regions of the LOW slope gradients overlap in crosslinked laminin concentration (see Figure 1) with “low” and “mid” regions of the HIGH slope gradients. For the three cancer cell lines (red = HEY-1, blue = OVCA433, and violet = SKOV-3.ip1), we found a smooth increase in speed with increasing metastatic potential for both the low and high gradient designs. This is similar as to what we found in our previous results for these cell types using constant concentration fibers of LN [36]. We also find that the speed increases for the SKOV-3.ip1 and OVCA433 cells with increasing gradient concentration, demonstrating a strong haptotactic response to the changing LN concentration.

Most of the comparisons for each cell response at each concentration range were statistically different for these two cell types (Table 1 and Table 2).

**Table 1:** *p* values from pair-wise *t*-tests of migration speeds of OVCA433 cells at low, medium, and high concentration regions of the low and high slope LN gradients. *n* = number of cells used in the analysis. In lieu of explicit *p* values, we denote “Y” (yes) as statistical differences corresponding to *p* < 0.05 to better visualize trends.

OVCA433	Low Slope Low *n* = 24	Low slope Medium *n* = 31	Low slope High *n* = 24	High slope Low *n* = 29	High slope Medium *n* = 40	High slope High *n* = 23
Low slope Low		Y	Y	Y	Y	Y
Low slope Medium			0.09	Y	0.29	Y
Low slope High				Y	0.38	Y
High slope Low					Y	Y
Highslope Medium						Y

**Table 2:** *p* values from pair-wise *t*-tests of migration speeds of SKOV-3.ip1 cells at low, medium, and high concentration regions of the low and high slope LN gradients. *n* = number of cells used in the analysis. “Y” corresponds to *p* < 0.05.

SKOV-3.ip1	Low Slope Low *n* = 12	Low Slope Medium *n* = 19	Low Slope High *n* = 15	High Slope Low *n* = 11	High Slope Medium *n* = 17	High Slope High *n* = 17
Low slope Low		Y	Y	Y	Y	Y
Low slope Medium			0.71	0.96	Y	Y
Low slope High				0.74	Y	Y
High slope Low					Y	Y
High slope Medium						Y

The IOSE cells (green) also displayed a strong migration speed response to the gradients, where the speed increased with increasing concentration, and also showed a strong dependence of the slope (statistics in Table 3).

**Table 3:** *p* values from pair-wise *t*-tests of migration speeds of IOSE cells at low, medium, and high concentration regions of the low and high slope LN gradients. *n* = number of cells used in the analysis. “Y” corresponds to *p* < 0.05.

IOSE	Low Slope Low *n* = 24	Low Slope Medium *n* = 31	Low Slope High *n* = 24	High Slope Low *n* = 29	High Slope Medium *n* = 40	High Slope High *n* = 23
Low slope Low		Y	Y	0.42	0.04	Y
Low slope Medium			Y	0.06	0.97	Y
Low slope High				Y	Y	0.29
High slope Low					0.10	Y
High slope Medium						Y

For example, the speed at the high concentration region of the low gradient was higher than the speeds at the low and medium regions of the high slope gradient. In our previous study of constant concentration LN fibers, the IOSE had slower migration speeds than the cancer cells [36]. However, here these cells were faster than the OVCA433 and SKOV-3.ip1 cells. A possible explanation lies in the relative size of these cells. They have much larger spread area than the OVCA43 and SKOV-3.ip1 cells (~3500 *vs.* ~2000 µm^2^) [36] and experience a larger range of concentration, and the slope of the gradients may play a larger role in the observed behavior.

Similar to our previous results, the highly metastatic HEY-1 cells had the highest migration speed [36]. In contrast to the other cells, the highly metastatic HEY-1 cells showed similar speeds at all concentration regions (see Table 4).

**Table 4:** *p* values from pair-wise *t*-tests of migration speeds of HEY-1 cells at low, medium, and high concentration regions of the low and high slope LN gradients. *n* = number of cells used in the analysis. “Y” corresponds to *p* < 0.05.

HEY-1	Low Slope Low *n* = 21	Low Slope Medium *n* = 25	Low Slope High *n* = 30	High Slope Low *n* = 25	High Slope Medium *n* = 21	High Slope High *n* = 26
Low slope Low		Y	0.32	Y	0.48	Y
Low slope Medium			0.64	0.78	0.19	0.36
Low slope High				0.52	0.66	0.26
High slope Low					0.12	0.5
High slope Medium						Y

**Figure 4:**
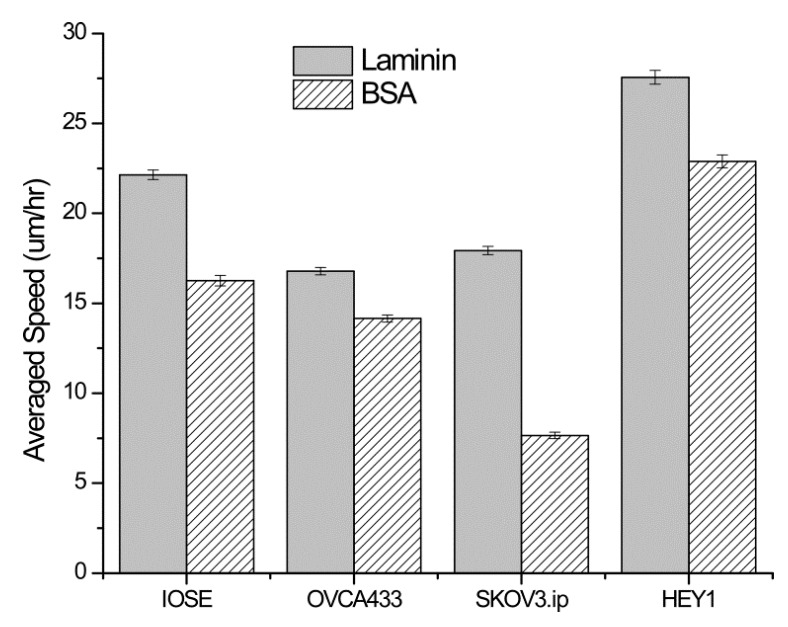
Average migration speed of the four cell lines on LN and BSA gradients made from the same parameters for the high slope design. All cells migrated with statistically faster speeds on the LN, demonstrating the importance of both ECM and contact cues. Error bars represent standard error. Data was analyzed from 40–100 cells in each case.

This independence of migration speed on LN concentration could suggest the migration for these cells is governed by contact guidance, rather than ECM binding cues. To investigate this idea, we performed analogous speed measurements on the same cell lines migrating on the analogous gradients created from BSA, which only provides contact guidance in the absence of ECM cues. Figure 4 shows the comparison of the migration speeds from each cell line on the high slope LN and BSA gradients (high concentration region). In this analysis the speeds were not parsed by concentration regions but determined over the entire length of the laminin and BSA gradients. All the cell types migrated faster on the LN than the BSA gradient, where the differences were all significant (*p* < 0.05). This is strong evidence that LN ECM cues (presumably through integrin binding) in conjunction with contact guidance contribute to the observed migration speed. We previously measured the migration of these cells on self-assembled monolayers and found random, slower migration than that observed on either LN or BSA constant concentration fibers, showing the ECM cues in conjunction with topographic cues result in faster migration than either alone [36].

**Figure 5:**
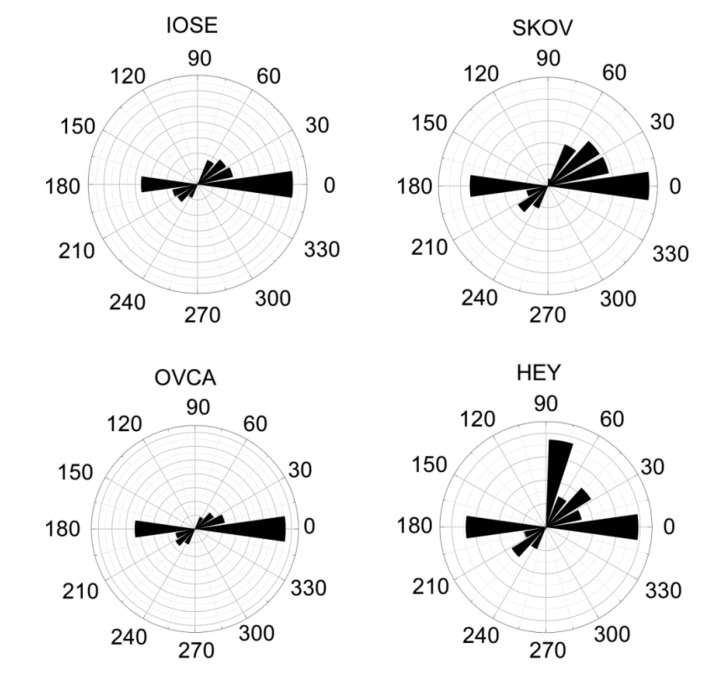
Directed migration of the four cells lines along the fiber axis of the high slope gradient shown as polar plots. The IOSE, OVCA433, and SKOV-3.ip1 cells had similar responses of directed migration based on the Rayleigh test and the pairwise *U*^2^ Watson test. The HEY-1 cells did not pass the Rayleigh test, indicating a random distribution and were different from the other cells based the pairwise *U*^2^ Watson test. The slight asymmetries are a computational artifact.

To further investigate the migration dynamics, we examined the directed migration by measuring the trajectories of each cell type relative to the gradient. The data for each line is shown as a polar plot in Figure 5. The IOSE, OVCA433, and SKOV-3.ip1 display similar behavior, where the migration vector is predominantly along the gradient axis. The polar distribution in each case passes the Rayleigh test, showing a statistical directionality. The Watson *U*^2^ tests between these cell types were not different. Together, this shows that the cells respond in a well-defined similar way to the gradient, and is consistent with the haptotactic response of each in Figure 3. The data for the HEY-1 cells was markedly different. We observed more of a random distribution, which did not pass the Rayleigh test. Moreover, the Watson *U*^2^ test was different relative to the other cells.

### 3.3. Cell Alignment on LN and BSA Gradients

We measured the resulting alignment of the four cell lines on LN gradients of the same low and high slope designs used for migration speeds. This was performed over the entire gradient as well as for three regions, which we again denote low, medium and high concentration, where these correspond to regions between 200–400 microns, 400–600 microns, and 600–800 microns, respectively. Images were selected at regular intervals over the 48 h observation time for this analysis. Cells were modeled as ellipses and the alignment was plotted as the angle between the long axis of the cell and the axis of the gradient [35]. The numbers of cells analyzed for each range were between 50 and 100.

**Figure 6:**
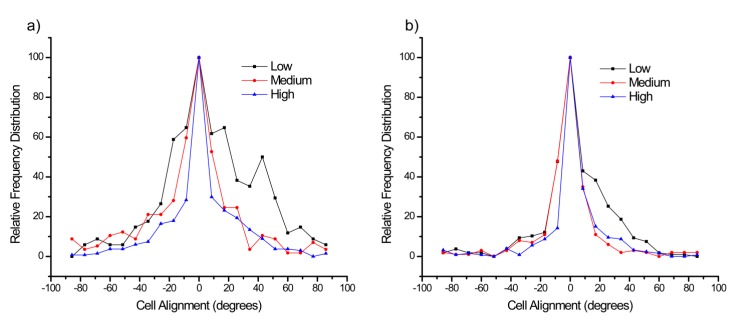
Histograms of the alignment of OVCA433 cells on the low (**a**); and high (**b**) LN gradients relative to the fiber axis. The alignment is further broken down into low, medium, and high concentration regions of the respective gradient. The alignments were all peaked at 0°, *i.e.*, parallel to the gradient but increased LN concentration resulted in narrower distributions until a saturated response was observed.

Figure 6a,b shows the distribution of angles of the OVCA433 cells for the lower and higher slope gradients over the three respective concentration regions. Here the data for each concentration was normalized to its own maximum to better observe the distribution. We found that for the lower gradient, each response was centered about 0 degrees, *i.e.*, parallel to the gradient direction, where cells on higher concentration regions had a tighter distribution in the orientation. This may arise due to the increased number of binding sites. In the higher slope gradient, the distribution continued to narrow, where the medium and high concentration regions had similar distributions. This is indicative of a saturated response, where increased binding sites did not affect the alignment. The distributions for the other cell types were also centered about 0 degrees, and in general decreased with increasing LN concentration. The respective widths of the distribution in terms of circular standard deviation are shown in the Table 5. For all cell lines, using the Watson *U*^2^ multisample test, the alignment for the regions on the low slope gradient was statistically broader than the medium and high concentration regions on the high slope gradient. This indicates that small changes in concentration have a fairly large effect on the alignment.

**Table 5:** Circular standard deviations of the four cell lines on the low, medium, and high concentration regions of the low and high slope gradients. The distributions narrowed at higher concentrations for all cell lines.

	LOW–Low	LOW–Med	LOW–High	HIGH–Low	HIGH–Med	HIGH–High
IOSE	38.3°	32.2°	23.7°	30.5°	27.4°	27.3°
OVCA433	32.2°	30.0°	24.3°	23.1°	21.2°	20.9°
SKOV-3.ip1	35.0°	32.6°	22.6°	30.5°	27.8°	25.9°
HEY-1	38.0°	32.0°	29.6°	35.7°	28.8°	28.4°

**Figure 7:**
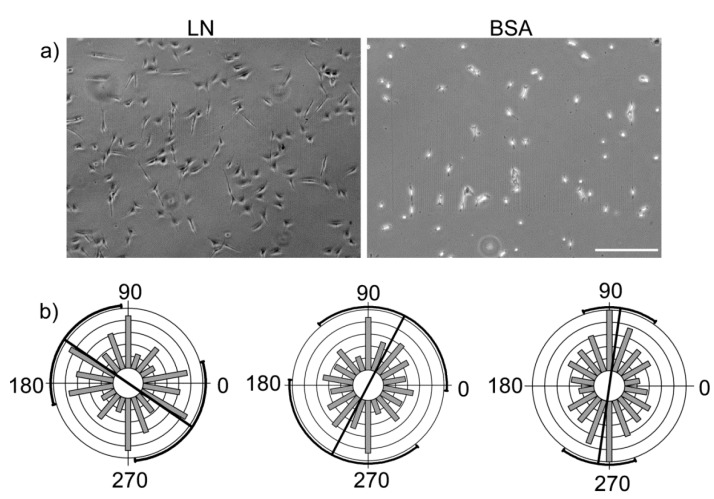
(**a**) Representative phase contrast images of OVCA433 cells on LN (**left**) and BSA (**right**) gradients 12 h after seeding. The cells are better spread on the LN gradient. Scale bar = 200 microns. (**b**) Polar plots of the orientation of the OVCA433 cells on the low, medium, and high slope ranges of the BSA gradient, showing a random distribution in each case. Polar plots are used for better visualization of the random distribution.

To compare the effect of contact guidance *vs.* ECM cues, the alignment response on a BSA high slope gradient from the same design parameters as in LN was also measured. Representative phase contrast images of IOSE cells on LN and BSA gradients at the same time (12 h post-seeding) are shown in Figure 7a. We found that the OVCA433 cells on LN were more spread and aligned at the same 12 h time point. This was also observed for the other cell types. The distribution of orientations for OVCA433 cells on the low, medium and high regions of the BSA gradient are shown in Figure 7b. We observed a more random distribution of alignment angles for this cell type as well as the others on the BSA gradient, where, in all cases, the responses did not pass the Rayleigh test and had a random distribution of orientations. Thus we concluded that the LN gradient provides operative ECM cues resulting in cell alignment along the axis of the gradient.

### 3.4. Cytoskeletal Alignment

**Figure 8:**
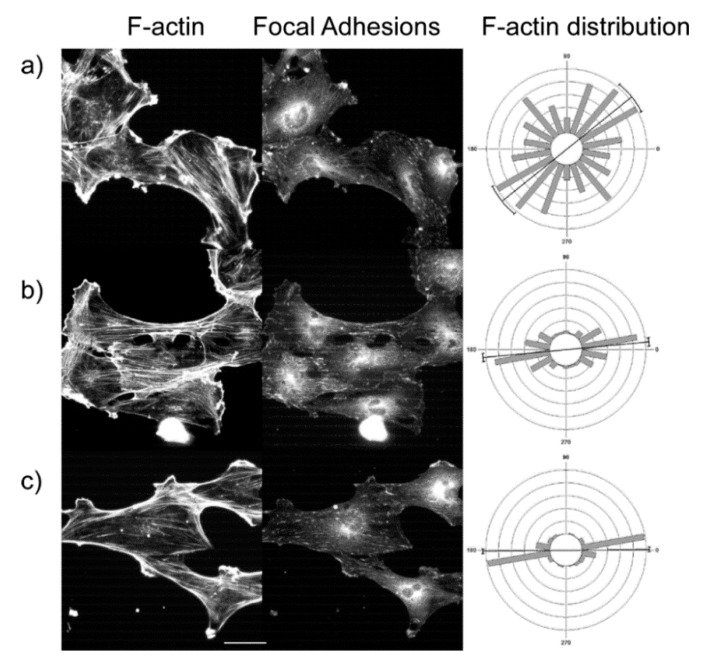
Representative cytoskeleton images (left = phalloidin; middle = anti-vinculin) for SKOV-3.ip1 cells on the LN high slope gradient for (**a**) low concentration; (**b**) medium concentration; and (**c**) high concentration regions. Scale bar = 40 microns. The right column is the f-actin angular distribution relative to the fiber axis, showing an increase in stress fiber alignment at higher LN concentration. The focal adhesion localization on the LN similarly increased. The statistical analysis for stress fibers and focal adhesions is given in Table 6 and Table 7, respectively.

We measured the response of the cytoskeleton of the cell lines on the low slope gradient. Cells were fixed and stained for actin stress fibers and focal adhesion as described in the Methods. HEY-1 cells, as reported previously do not display distinct stress fibers or focal adhesions [36] and are not included in this analysis. We determined the angular distribution of stress fibers relative to the gradient axis over low, medium, and high concentration ranges as was done in the previous section. Representative fluorescence images of SKOV-3.ip cells and the resulting angular distributions over multiple cells (300–500 measured stress fibers in each case) are shown in the left and middle columns, respectively, in Figure 8. Here we grouped the actin orientation per region of the gradient rather than comparing the stress fibers within single cells and the statistical comparison was then performed between these groups.

The results for the three cell lines and three concentration ranges in terms of mean vector and circular standard deviation are summarized in Table 6. We found that at low concentration, the distribution was essentially random and became highly aligned (*i.e*., towards 0°) at medium and high concentration, although there is a distribution of angles around the mean. For each cell, the Pair-wise Watson *U*^2^ tests showed that the distribution at each concentration was statistically different (*p* < 0.001). These alignment results are also consistent with the ECM cues guiding the alignment of the f-actin cytoskeleton. This is further consistent with these cues guiding the cell orientation (Section 3.3).

**Table 6:** F-actin mean angle and distribution relative to the long axis for the cells lines on the low slope gradient. The average angle for three concentration regions of each cell type was statistically different. *n* = 300 to 500 stress fibers per region and the errors are standard deviations.

	Low	Medium	High
IOSE	70.6 ± 67.6	6.0 ± 35.2	1.4 ± 26.7
OVCA433	54.1 ± 55.8	3.9 ± 38.0	1.0 ± 23.9
SKOV-3.ip1	55.7 ± 55.7	5.4 ± 25.6	0.6 ± 15.07

We also investigated the distribution of focal adhesions as a function of LN concentration. Representative anti-vinculin immunofluorescence images of the focal adhesions for SKOV-3.ip1 cells are shown in the right column of Figure 8 next to the corresponding images of the actin stress fibers and angular distribution. For all concentrations, some punctuate focal adhesions are associated with the LN features. In analogy with the stress fiber analysis, these data points were grouped and analyzed based on regions along the gradient rather than by within individual cells. We measured the probability that focal adhesions were located on the LN gradients as opposed to spaces between the patterns. The data for IOSE, OVCA433, and SKOV-3 cells are tabulated in Table 7, where a value of 1 corresponds to equal probability of the adhesions being on the LN and the BSA background between in linear gradient, and higher values are the increased probability of adhering to the gradient. The theoretical limits are then 0 and infinity for having none and all of the adhesions on the gradients, respectively. For the IOSE and SKOV-3.ip1 cells, the probability increased with concentration (*p* < 0.01). The OVCA433 values were statistically similar but all statistically greater than unit probability.

**Table 7:** Ratio of focal adhesion overlap on LN gradients *vs.* BSA background for the cells lines on the low slope gradient. The ratios for the IOSE and SKOV-3 cells were different at the three concentration regions. The OVCA433 data were not different from each other were all greater than unity. Focal adhesions from 16–20 cells are analyzed per region and the errors are mean error.

	Low	Medium	High
IOSE	0.94 ± 0.05	1.33 ± 0.08	1.54 ± 0.07
OVCA433	1.10 ± 0.11	1.12 ± 0.09	1.25 ± 0.09
SKOV-3.ip1	1.06 ± 0.06	1.40 ± 0.05	1.57 ± 0.06

While these preferred factors may seem low, the relative area covered by the LN gradient *vs.* the BSA background is less than 10%, thus, based on area, the preference can be considered to be more pronounced. Moreover, it was not expected that the focal adhesions would be solely located on the LN as the BSA monolayer is not a repulsive background and the cells will eventually form new adhesions, especially as new matrix is formed. This is a different scenario than sometimes reported in the literature where islands or stripes of attractive regions were placed against a cell-repulsive background, and the focal adhesions are expressed solely on the fabricated regions by default [41,42].

## 4. Discussion

### 4.1. Comparisons with Other Methods

Most of the prior research utilizing photo-patterned laminin gradients was in neuronal biology and investigated if haptotaxis was operative in promoting axonal growth [20,23]. One of the most detailed studies of cell migration and polarity created and utilized covalently patterned laminin gradients of various different slopes and concentration via microfluidic channels [21,22]. They found increasing migration speeds with increasing surface concentration. We did not see the bell shaped curve in terms of migration speed as a function of concentration seen in [21,22] and in the seminal report by Lauffenberger and Horwitz [43]. However, it is difficult to make direct comparisons of concentrations as these previous studies were on two dimensional surfaces, whereas the gradient constructs here are 2.5D, *i.e.*, they have well defined but finite height (1–2 microns). Additionally, the gradients in these previous reports were comparable in length to those used here but were comprised of stripes of hundreds of microns in width. It is not readily possible to use microfluidic approaches to create the submicron sizes achievable by MPE fabrication. This is an important consideration as cells respond to morphological features on this size scale, especially in terms of adhesion as focal adhesions are on the order a few hundred nanometers in width. We do note that the microcontact printing approaches have better scalability and throughput as it is much simpler to make multiple copies from the same design.

Perhaps the largest advantage of the MPE fabrication approach is that it allows the decoupling of migration dynamics arising purely from topography, *i.e.*, contact guidance and the combined effects of binding cues and topographic cues presented by ECM proteins. These effects are believed to be important in cancer growth and metastasis. For example, by studying migration in 3D collagen gels, contact guidance was implicated in invasion of breast carcinoma [44,45]. Such collagen gels are powerful tools as stromal models, however the polymerization dynamics are highly sensitive to temperature and pH and the resulting fiber sizes and distributions are not highly reproducible. In contrast, MPE fabrication affords a *de novo* approach to build simple highly reproducible models to study migration dynamics under well-controlled conditions.

The ability to create well-defined gradients will add to the toolbox to study cancer biology. Haptotaxis has long been suggested to be important in metastasis [46] but it has not been well-studied, perhaps due to the lack of adequate technologies. For example in 1987 Liotta performed a biochemical analysis of the laminin receptor in tumor cell migration [47]. The approach here allowed the study of adhesion/migration dynamics of ovarian cancer cells on laminin of well-defined concentration through the combination of nano/microfabrication and live cell imaging. Moreover, we used 2.5D structures, which are more biomimetic in terms of dimension than flat 2D printed surfaces. The importance of non 2D geometries was previously demonstrated by Yamada [41], where they showed that even 1D protein stripes on 2D surfaces were effective in driving cell migration. The 2.5D patterns here have smaller dimension in width (~700 nm) that possible through their approach and more nearly approximate the size of cell binding sites.

### 4.2. Relevance to Ovarian Cancer Cell Biology

We found the most of the cell types, with the exception of the highly metastatic HEY-1 cells migrated faster on higher concentrations of LN and displayed highly directed migration along the gradient axis (Figure 3). All the cells displayed a higher degree of alignment at higher LN concentration until a saturated response was observed. Taken together, these effects demonstrate the migration dynamics result from a combined effect of ECM binding cues from the LN and also contact guidance through the submicron topography features. In strong contrast, all cell types migrated slower on BSA gradients of the same design which only provided pure contact guidance. The alignment of the cells on BSA gradients was also randomly distributed (Figure 7b), further pointing to the importance of ECM cues on the cell dynamics.

We next consider why the highly metastatic HEY-1 cells displayed very different behavior in terms of migration speed and directed migration compared to the other cell types. Perhaps the answer lies in the observation that these cells have different cytoskeletal organization, specifically they do not have clear stress fibers or discrete focal adhesions. These cells migrate faster than the other cell types with less directed migration in a largely concentration independent manner. Yet the HEY-1 cell migration on LN was faster than on BSA gradients, precluding a pure contact guidance mechanism. The rapid migration may arise from the combination of the flexible cytoskeletal properties (*i.e*., no stress fibers and branched morphology) and the contact guidance provided by the laminin fibers. This suggests these cells migrate in an integrin-independent mechanism or perhaps the turnover is rapid and averaged out when the cells are fixed and stained. Similar integrin-independent ameboid-like migration was observed by Friedl for t-lymphocytes in 3D collagen gels [48,49]. In terms of *in vivo* behavior, the low density of focal adhesions may result in exfoliation from the surface of the ovary and provide part of the metastasis mechanism, which has been observed clinically [50].

This study also investigated the roles of local concentration and slope of gradients on cell migration dynamics using two gradient designs. This is an open question in cell biology, and using these models, we found that the answer is not easily generalizable. The branched HEY-1 cells migrated at similar speeds at almost all concentrations. The IOSE cells, which are larger than the other cell types, had migration speeds, which depended on both the slope and concentration (see Figure 3). For the smaller but more regular shaped SKOV-3.ip1 and OVCA433 cells, the local concentration was the dominant factor. Thus, the cell shape and size relative to the dimensions of the gradient all factor into the haptotaxis dynamics. Overall, we found that relatively small changes in concentration and slope have a large effect on the migration dynamics.

We lastly note that while background adsorption of matrix molecules from serum to the surface is a potentially confounding factor, especially on the neutral BSA gradients we do not believe it played a large role in this study. This is evidenced by the difference in morphology shown for IOSE cells on BSA and LN gradients in Figure 8a at the same 12 h time point post seeding. Further, all cell types displayed this behavior.

## 5. Conclusions

We used MPE fabricated gradients of LN to study haptotaxis of a series of ovarian cancer cells. Using these 2.5D models of the basal lamina, we found that the increased LN concentration increased migration speed and alignment of the cells along the linear axis of the gradients. Both these metrics were enhanced on LN compared to BSA gradients of the same design, demonstrating the importance of both topographic and ECM cues on the adhesion/migration dynamics. Using two different gradient designs, we addressed the question of the roles of local concentration and slope and found that the specific haptotactic response depends on the cell phenotype and not simply the gradient design. This work is a necessary step in studying haptotaxis in more complete 3D models of the tumor microenvironment.
